# A data-driven analysis on the mediation effect of compartment models between control measures and COVID-19 epidemics

**DOI:** 10.1016/j.heliyon.2024.e33850

**Published:** 2024-06-29

**Authors:** Dongyan Zhang, Wuyue Yang, Wanqi Wen, Liangrong Peng, Changjing Zhuge, Liu Hong

**Affiliations:** a School of Mathematics, Sun Yat-Sen University, Guangzhou, Guangdong, 510275, PR China; b Department of Mathematics, School of Mathematics, Statistics and Mechanics, Beijing University of Technology, Beijing, 100124, PR China; c Beijing Institute of Mathematical Sciences and Applications, Beijing, 101408, PR China; d College of Mathematics and Data Science, Minjiang University, Fuzhou, 350108, Fujian, PR China

**Keywords:** COVID-19, Compartment model, Mediation effect, Policy evaluation, Linear and nonlinear regressions

## Abstract

By collecting various control policies taken by 127 countries/territories during the first wave of COVID-19 pandemic until July 2nd, 2020, we evaluate their impacts on the epidemic dynamics quantitatively through a combination of the multiple linear regression, neural-network-based nonlinear regression and sensitivity analysis. Remarkable differences in the public health policies are observed across these countries, which affect the spreading rate and infected population size to a great extent. Several key dynamical features, like the normalized cumulative numbers of confirmed/cured/death cases on the 100th day and the half time, show statistically significant linear correlations with the control measures, which thereby confirms their dramatic impacts. Most importantly, we perform the mediation analysis on the SEIR-QD model, a representative of general compartment models, by using the structure equation modeling for multiple mediators operating in parallel. This, to the best of our knowledge, is the first of its kind in the field of epidemiology. The infection rate and the protection rate of the SEIR-QD model are confirmed to exhibit a statistically significant mediation effect between the control measures and dynamical features of epidemics. The mediation effect along the pathway from control measures in Category 2 to four dynamical features through the infection rate, highlights the crucial role of nucleic acid testing and suspected cases tracing in containing the spread of the epidemic. Our data-driven analysis offers a deeper insight into the inherent correlations between the effectiveness of public health policies and the dynamic features of COVID-19 epidemics.

## 1 Introduction

Since its first emergence at the end of 2019, the Corona Virus Disease 2019 (COVID-19) has swept across more than 222 countries and territories around the world. Until May 3rd, 2023, the World Health Organization (WHO) has reported over 750 million confirmed cases and nearly 7 million fatalities [1]. The actual death toll from COVID-19 is likely to be threefold higher than the official counts.

During its spreading, the primitive novel coronavirus, SARS-CoV-2 has undergone multiple mutations, resulting in variants like Delta and Omicron with increased transmission rates and ability of immune escape [2]. A number of control strategies were implemented globally to mitigate the pandemic, including but not limited to vaccination, quarantine, contact tracing, travel limitations, border closures, as well as mandates for mask-wearing and social distancing.

On May 5th, 2023, WHO declared an end to the global COVID-19 public health emergency. This means a significant transition from critical emergency response activities to long-term sustained COVID-19 disease prevention, control and management. Therefore, it is an opportune time to retrospectively analyze and assess the efficacy of the public health policies during the pandemic.

In the literature, there are extensive studies on COVID-19 containment policies [[3], [4], [5], [6], [7], [8], [9]]. For example, it has been revealed that the implementation of non-pharmaceutical interventions (NPIs), especially those implemented at the early stage of the epidemic, can effectively inhibit the development of the epidemic [10,11]. According to Refs. [12,13], school closures played an important role in the emergence of a pandemic. This conclusion was further confirmed by a study of the outbreak in the United States [14], which showed that school closures can reduce COVID-19 morbidity and mortality by about 60 %. Wong et al. [15] examined the association of NPIs with reduced burden of COVID-19, and found that closing schools and workplaces has an impact on mitigating the disease. Xiang et al. [16] revealed that improving quarantine and reporting rates and using protective masks are crucial for epidemic prevention and control. Literature [11,17] suggested that the lockdown, restrictions on social gatherings, remote work, and school closures are the most effective NPIs.

Meanwhile, Balmford et al. [3] made cross-country comparisons and showed that policy interventions, rather than the socioeconomic factors, determine the majority of variations in death rates of COVID-19 among OECD members. Unruh et al. [18] compared COVID-19 health policy responses in Canada, Ireland, UK and US, and concluded that the health system capacity, governance and political leadership all shaped country responses. To evaluate and compare the effectiveness of various policies, Koh et al. [19] regressed some physical distancing measures, including international travel controls, restrictions on mass gatherings, and lockdown, on the time-varying reproduction number.

A lot of efforts have been dedicated to a comprehensive collection of NPIs. Among them, Hale et al. [20] constructed an influential database, called the *Oxford COVID-19 Government Response Tracker* (OxCGRT), to capture the government policies for over 180 countries/territories. With 19 policy indicators including containment and closure, economic response and health systems, OxCGRT enables further research that integrates the policy responses with epidemiological indicators. There are alternative datasets, such as ECDC-JRC [21], HIT-COVID [22], CCCSL [23], CoronaNet COVID-19 [24], that differ slightly from the OxCGRT database in terms of the policy implementation time, policy classification, and etc.

Based on the above policy databases, Lewnard and Lo [25] found that combined interventions were most effective only when isolation, school closure, and workplace distancing were included. When considering the four measures of mandatory wearing of masks in public places, isolation or quarantine, social distancing, and traffic restrictions, implementing two or more actions at the same time would be of a strategic priority for the containment of COVID-19 [26]. By using the weighted random effects regression and univariate and multivariate analyses, Jüni et al. [27] revealed a strong correlation between epidemic growth and the number of public health interventions implemented, such as school closures, mass assembly restrictions, and social distancing measures. However, the simultaneous implementation of multiple measures will place a great burden on the finance.

With the help of mathematical models, we can conduct more quantitative analyses. For example, Tang et al. [28] revealed the efficacy of public health interventions and detection rates in countries, like China and South Korea. As to municipal policy responses to COVID-19, Armstrong et al. [29] used a survey in Canada to measure the aggressiveness of responses. This latent variable was found to be closely related to municipal population size and COVID-19 cases. Zhou et al. [30] tailored the SEIR model to China's “dynamic zero-COVID policy”, underscoring the efficacy of stringent containment strategies. For a survey of various compartment models for COVID-19 dynamics, see e.g. Ref. [31].

In addition to ODE-based compartment models, more sophisticated models have also been employed to study the transmission processes. For example, several works modeled the COVID-19 dynamics by fractional differential equations when the memory effects, heavy-tailed effects or long-range interactions were non-negligible [[32], [33], [34], [35]], or when the lockdown measures [36,37] or the influence of social media was considered [38]. Jan and Boulaaras et al. studied the transmission dynamics of viral infection with effective control policies via fractional derivative [34,35]. Comprehensive analyses about the uniqueness, existence and local asymptotic stability of the model solution with wide applications to chikungunya virus infection, hand–foot–mouth disease and typhoid fever were carried out [[39], [40], [41]]. Besides, the infection of epithelial cells by the SARS-CoV-2 could also be modeled by fractional differential equations [42]. On the other hand, the delay differential equations were often employed to incorporate the delay effects caused by vaccination, immunity or the latent period of an infectious disease [43,44]. The introduction of time delay, to some extent, provided a more accurate representation of disease transmission dynamics with fewer compartments compared with those by ordinary differential equations [45]. It could also reduce the number of parameters, making the models more robust and reliable [46]. Moreover, the delay effects and the vanishing of immunity could account for the multi-wave dynamics of COVID-19 outbreaks even without considering the emergence of new variants [47].

As the transmission of COVID-19 is mainly based on the person-to-person contact, complex networks are essential for accurately characterizing the disease transmission pattern. During the COVID-19 pandemics, many theoretical and numerical approaches have been developed to analyze and simulate the epidemic dynamics on complex networks [[48], [49], [50], [51], [52], [53]]. Unlike the spatial continuous models, the network-based models can well capture the discrete interactions among individuals. This makes them particularly suitable for investigating scenarios beyond the scope of spatial continuous models, such as the individual human behavior under different policies [48], diverse contact patterns [49], geographic locations [54], etc. Furthermore, to model the epidemic dynamics influenced by information flow [53,55] or under realistic situations, higher-order networks, multi-layer networks or temporal networks are natural choices [56,57].

Motivated by previous studies, our current research aims to quantify the intrinsic correlations between public health policies and epidemic dynamics through a combination of several novel statistical methods, thus enhancing policy design and implementation for future pandemics. Additionally, we hope to elucidate the significant role of compartment models in the study of epidemics from a new perspective – its mediation effect, which has not been explored yet in this field to the best of our knowledge, but is crucial for understanding the intricate linkages between control measures and COVID-19 transmission dynamics.

Particularly, in this study we try to address the following key research questions, i.e.

• How do the public health policies and their implementation intensities adopted by each country affect the spreading dynamics of COVID-19 epidemics?
• Do some control policies act more effective than the others? What are they?
• From a statistical aspect, does the inclusion of compartment models really provide a better understanding on the intrinsic relations between control measures and the epidemic dynamics?

This work is structured as follows. Section 2 details a collection of epidemic data and public health policies of 127 countries/territories. A novel compartment model for epidemics, the mediation analysis with structural equation modeling, as well as methods on clustering, correlation and regression analyses are briefly introduced too. In Section 3, our main results including the classification of control measures, the clustering of 127 countries/territories, the spreading dynamics of COVID-19 characterized through compartment models, impacts of control measures on the spreading dynamics and the mediation effect of compartment models are reported separately. The conclusion and discussions are presented in Section 4.

## 2 Materials and methods

### 2.1 Data collection

To quantify the impact of public health policies on COVID-19 transmission dynamics, we collect data on 16 distinct control measures marked by discrete levels, for 127 countries/territories (see data file 1 in Supplementary). These measures were excused by governments up to July 7, 2020 during the initial wave of the epidemic. Although the provision of medical resources does not constitute a preventive or containment measure *per se*, it is included due to its substantial effect on the recovery and mortality of patients. Concurrently, we collate the official WHO data on daily new, recovered, and deceased COVID-19 case counts [58] (see data file 2 in Supplementary). For consistency, the epidemic onset in each country is defined as the date when the cumulative case count reached 100.

### 2.2 Mediation analysis with structural equation modeling

The mediation analysis elucidates the effect of an independent variable (*X*) on a dependent variable (*Y*) through intermediary variables known as mediators (*M*). This analytical framework is frequently utilized in applied disciplines, such as psychology and education, where it facilitates the understanding of complex causal structures [65]. In addition, the Structural Equation Modeling (SEM) enables the examination of multiple independent and mediator variables, both observed and latent, within a unified framework [66].

For our purpose, here we illustrate the basic idea of structural equation modeling for mediation analysis with multiple mediators operating in parallel. Without loss of generality, let *X*_1_ and *X*_2_ be two independent variables, *Y* be the dependent variable, and *M*_1_ and *M*_2_ be the mediators independent of each other. The SEM framework is represented by the following linear regression equations (1a), (1b), (1c), (1d),

(1a) $$Y=\tau_{1}X_{1}+\tau_{2}X_{2}+\varepsilon_{X}\text{,}$$

(1b) $$M_{1}=\eta_{11}X_{1}+\eta_{12}X_{2}+\varepsilon_{1}\text{,}$$

(1c) $$M_{2}=\eta_{21}X_{1}+\eta_{22}X_{2}+\varepsilon_{2}\text{,}$$

(1d) $$Y=\tau_{1}^{\prime }X_{1}+\tau_{2}^{\prime }X_{2}+\mu_{1}M_{1}+\mu_{2}M_{2}+\varepsilon_{Y}\text{,}$$

where *τ* ′s and *η*′s are linear regression coefficients and *ε*′s denote errors. Moreover, based on above formulas, the difference of the coefficients *τ*_1_ – *τ*_1_*′* (resp. *τ*_2_ – *τ*_2_*′*) is recognized as the total mediation effect of *X*_1_ (resp. *X*_2_) on *Y*, that is,

(2) $$\tau_{1}\;-\;\tau_{1}^{\prime }=\mu_{1}\eta_{11}+\mu_{2}\eta_{21},\tau_{2}\;-\;\tau_{2}^{\prime }=\mu_{1}\eta_{12}+\mu_{2}\eta_{22}\text{,}$$

where the constitutive term *μ*_*i*_*η*_*ij*_ represents the corresponding individual mediation effect from *X*_*j*_ to *Y* through *M*_*i*_ (*i, j* = 1*,* 2).

To conduct mediation analysis, each variable is centralized by subtracting its mean value. Bootstrapping is employed to generate a robust dataset for SEM estimation, and then the undetermined coefficients are estimated according to the dataset [66]. An acceptable model fit enables us to proceed to significance testing of mediation effects using the bias-corrected bootstrap confidence intervals. And the significance is inferred if zero is not within these intervals.

In the context of the SEIR-QD model, the four categories of policies are considered as independent variables (*X*_1_*,* · · ·*, X*_4_), representing the 16 control measures in Table 1. Log-transformed coefficients in the SEIR-QD model (*M*_1_*,* · · ·*, M*_5_) ≡ (log(*α*)*,* log(*β*)*,* log(*γ*)*,* log(*δ*)*,* log(*κ*)) are used as the mediator variables. The dependent variable *Y* is defined as one of six log-transformed epidemiological features, thereby characterizing the COVID-19 spreading dynamics, *i.e. Y* ∈ {log(*Q*_100_*/N*)*,* log(*R*_100_*/N*)*,* log(*D*_100_*/N*)*,* log(*t*_1*/*2_)*,* log(*t*_*lag*_)*,* log(*k*_*app*_)}. The “*lavaan*” package in R language [67] is used to perform our mediation analysis with structural equation modeling (see data file 3 in Supplementary).

**Table 1:**
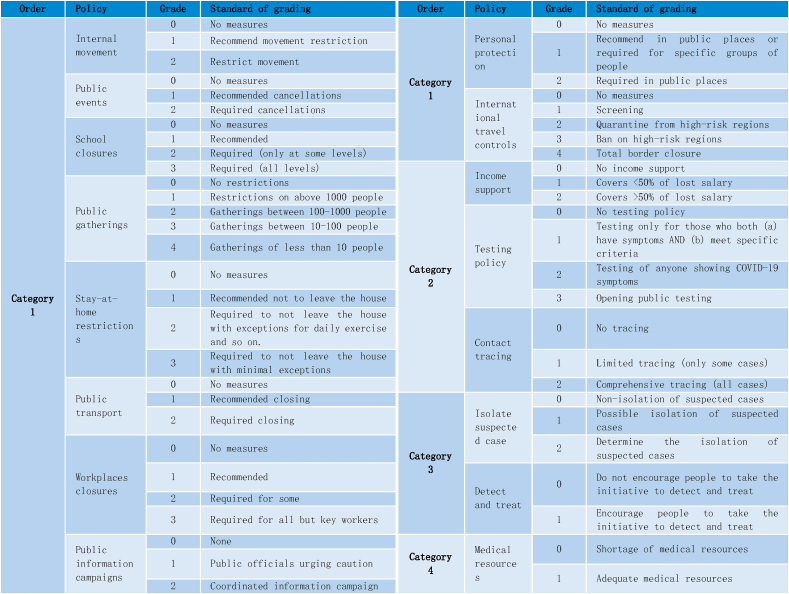
Table 1 Summary of public health policies taken by each country till July 7, 2020 during the first wave of COVID-19 pandemics. All 16 collected policies are classified into four categories. The strength of policies is characterized as ordinal data with 2–4 levels. Policy “School closures” in Category 1 is taken from Ref. [59]; other policies in Category 1 and policy in Category 4 from Ref. [60]; policies in Category 2 from Ref. [58]; policy “Isolate suspected case” in Category 3 from Refs. [[61], [62], [63], [64]].

### 2.3 Compartment models for epidemics

As a newly emerging highly infectious disease, COVID-19 exhibited distinctive characteristics compared to other infectious diseases, such as a higher proportion of asymptomatic carriers. Besides, unprecedented public health emergency responses were enacted globally during the pandemic's first wave, including the lockdown and mandatory quarantines, which have significantly altered the epidemic dynamics.

Considering the distinguishing features of COVID-19, the SEIR-QD model (Eq. (3a)-(3g), [68,69]) has demonstrated its capability in modeling the transmission dynamics of COVID-19 epidemic, and attracted extensive attentions [[70], [71], [72]]. This model extends the classic SEIR framework by integrating three additional compartments: one for protected individuals due to enhanced prevention measures, one for quarantined patients who are unable to infect susceptible individuals, and the last one for recovered individuals, distinct from the one for deceased cases.

In summary, the SEIR-QD model encompasses seven compartments – susceptible *S*(*t*), protected *P* (*t*), exposed *E*(*t*), infected *I*(*t*), quarantined *Q*(*t*), recovered *R*(*t*), and deceased *D*(*t*). Transitions between compartments are parameterized through six rates: *α*, *β*, *γ*, *δ*, *λ*, and *κ*. The corresponding differential equations read

(3a) $$\frac{dS\left(t\right)}{dt}=-\frac{\beta S\left(t\right)I\left(t\right)}{N}-\alpha S\left(t\right)\text{,}$$

(3b) $$\frac{dE\left(t\right)}{dt}=\beta \frac{S\left(t\right)I\left(t\right)}{N}\;-\;\gamma E\left(t\right)\text{,}$$

(3c) $$\frac{dI\left(t\right)}{dt}=\gamma E\left(t\right)-\delta I\left(t\right)\text{,}$$

(3d) $$\frac{dQ\left(t\right)}{dt}=\delta I\left(t\right)-\lambda Q\left(t\right)-\kappa Q\left(t\right)\text{,}$$

(3e) $$\frac{dR\left(t\right)}{dt}=\lambda Q\left(t\right)\text{,}$$

(3f) $$\frac{dD\left(t\right)}{dt}=\kappa Q\left(t\right)\text{,}$$

(3g) $$\frac{dP\left(t\right)}{dt}=\alpha S\left(t\right)\text{,}$$

in which *N* represents the total population.

The ordinary differential equations are solved by the Euler method. To reliably estimate the parameters in equations (3a), (3b), (3c), (3d), (3e), (3f), (3g), a critical step is to deal with the issue of non-identifiability, which is common in the study of compartmental models of epidemic dynamics due to both data limitations and model complexity, as high-lighted in many previous studies [[73], [74], [75], [76]]. Importantly, although parameter estimation may be non-identifiable, the basic reproduction number *R*_0_ derived from these estimations often remains robust [73,75]. Accordingly, we fit the daily new cases, daily new deaths, and daily new cured cases, as well as the cumulative infected cases, to ensure both the dynamic trends and the final size are well captured. Utilizing the ‘‘*lsqnonlin’’* function in MATLAB 2021b [77] for the nonlinear least squares optimization, we calibrated the SEIR-QD model parameters based on the collected epidemic data. This procedure is repeated for a thousand times with respect to diverse initial values, in order to minimize the influence of non-identifiability and obtain both stable and reliable parameters. The estimated optimal parameters are listed in Data-file-4 in the Supplementary Materials.

### 2.4 Data clustering

Considering the vast diversity in COVID-19 responses across nations, it is necessary to group the countries/territories prior to further analysis. To this end, the K-means clustering algorithm is applied, with the number of clusters setting to be 4 and the Euclidean distance as the similarity metric for data samples.

To ascertain the statistical significance of differences between clusters, we employ the Kruskal-Wallis rank sum test. This non-parametric test replaces observed values with their ranks for variance analysis. With *M* samples across *k* groups, where the *i*'th group contains *m_i_* samples and $M=\sum_{i=1}^{k}m_{i}$, we rank all samples in an ascending order. Data with identical values receive the average of their ranks. A significant Kruskal-Wallis *H* statistic,

(4) $$H=\frac{12}{M\left(M+1\right)}\sum_{i=1}^{k}\frac{R_{i}^{2}}{m^{2}}-3\left(M+1\right)∼X_{k-1}^{2}\text{,}$$

exceeding the threshold of the chosen significance level, indicates a significant variation among the groups, with *R*_*i*_ denoting the sum of ranks.

### 2.5 Correlation analysis

The Pearson's correlation coefficient quantifies the linear relationship between two variables. Given n observations of variables A and B, the coefficient is defined as:

(5) $$\rho \left(A,B\right)=\frac{1}{n-1}\sum_{i=1}^{n}\left(\frac{A_{i}-\mu_{A}}{\sigma_{A}}\right)\left(\frac{B_{i}-\mu_{B}}{\sigma_{B}}\right)\text{,}$$

with *μ*_*A*_ and *σ*_*A*_ representing the mean and standard deviation of *A*, and similarly for *B*. This method can be extended to multiple variables. We utilize the *Corrcoef* function in Matlab for computation and the *heatmap* function for graphical representation.

### 2.6 Multiple linear and nonlinear regressions

To capture the epidemic's complexity, we introduce six dynamic features: the normalized cumulative counts of confirmed, recovered, and deceased cases on day 100 (*Q*_100_*/N*, *R*_100_*/N*, *D*_100_*/N*), the half time *t*_1*/*2_, lag time *t*_*lag*_, and apparent spreading rate *k*_*app*_.

Our current study probes the impact of control measures on epidemic transmission using both linear and nonlinear regression approaches. The multiple linear regression is performed with the least squares method, augmented by the stepwise and principal component regressions using the *regress*, *stepwise*, and *pcacov* functions in Matlab.

The nonlinear regression employs a neural network model, the multilayer perceptron (MLP) to be exact, with an input layer and a hidden layer of 100 nodes using the *ReLU* function, and an output layer with the *Sigmoid* function. Inputs are 16 control measures, and outputs are six epidemic features. The network's loss function is the *L*_2_ norm of deviations between actual and predicted values, trained for 10000 epochs using the Adam optimizer with a learning rate of 10^*−*3^. The optimized batch size (set to be 10) enhances the efficiency of convergence.

## 3 Results

### 3.1 Global control measures are highly diverse during the first wave of the COVID-19 pandemic

The 16 control measures executed by 127 countries/territories analyzed in this study (detailed in Data collection and Table 1) exhibit complex inter-dependencies. For instance, stringent public gathering restrictions are often associated with school and workplace closures, necessitating an exploration of their correlations.

**Fig. 1:**
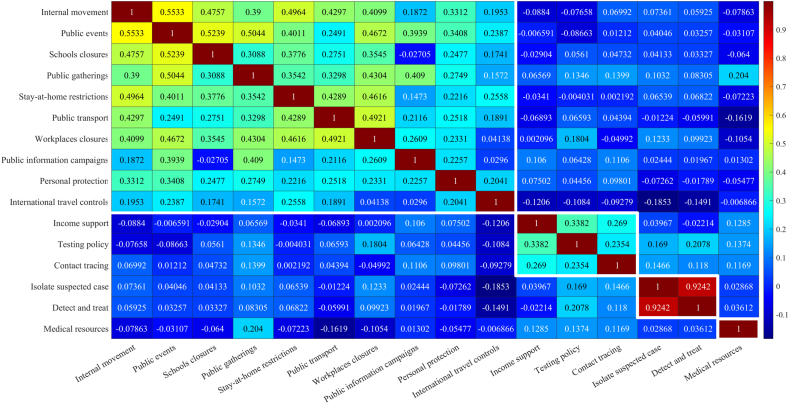
Heat map for correlations among control measures of 127 countries/territories. Each of 16 control measures is represented by a 127d vector, whose element gives the implementation strength of the policy in the corresponding country. Strong positive correlations are indicated by dark red, and weak correlations by dark blue, based on which we arrive at the four categories of policies taken in Table 1. (For interpretation of the references to color in this figure legend, the reader is referred to the Web version of this article.)

To this end, heat map is used for descriptive analysis on the control measures (Fig. 1), according to which, it becomes clear that the 16 control measures can be aggregated into four categories (Table 1). The first category includes transportation limitations and public space closures, which collectively reduce the number of susceptible individuals and contact rates. The second category includes policies on contact tracking and testing, impacting case detection. The third category refers to the pro-motion of testing and the implementation of quarantine measures on suspected cases, reflecting the efficacy of quarantine protocols. The last category reflects the status of medical resources. As early as 2006, Ferguson et al. [78] found that the effectiveness of social distancing, rapid case identification, and targeted prevention is similar, with school closures playing an important role in each scenario. This finding is consistent with our results on categorization.

**Fig. 2:**
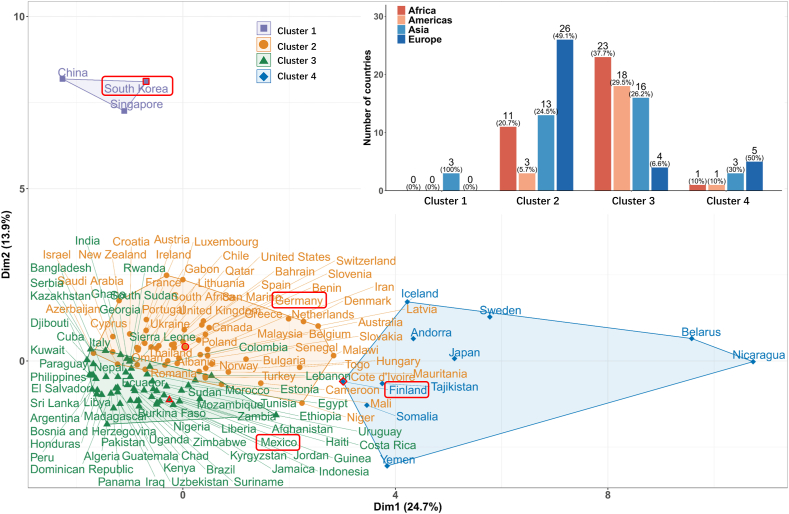
Clustering of 127 countries/territories based on their respective implementation intensity of control measures, whose data form is a 16d vector. Four clusters are identified by the K-means algorithm and marked in different colors. The t-SNE method is used for data visualization. The inset displays the continental distribution within each cluster, from which we can clear see that Cluster 1 is constituted by three Asian countries, the majority of Cluster 2 are countries in Western Europe, while most developing countries belong to Cluster 3. (For interpretation of the references to color in this figure legend, the reader is referred to the Web version of this article.)

On the other hand, control measures and their implementation intensity are dramatic different during the first wave of COVID-19 pandemic. By using the K-means method, countries are also clustered into 4 groups based on their own implementation strength of control measures. As visualized in Fig. 2, ***Cluster 1*** includes 3 Asian countries, China, South Korea, and Singapore, which takes strict control measures and keeps the pandemic well contained during the early 2020. ***Cluster 2*** includes 53 countries/territories, more than half of which are European countries. Most countries in this group have rich medical resources and take mediate-level measures of prevention and containment. According to Ref. [79], countries with lower risk awareness, younger populations, and more robust health care systems exhibit less pronounced adoption rates of policies, such as school closures and remote working. ***Cluster 3*** comprises 61 nations/territories, predominantly encompassing developing countries of the Third World with inadequate medical resources and less stringent control measures. ***Cluster 4*** includes the remaining 10 countries. This cluster is of greater diversity than the preceding three ones. Actually, we believe the inconsistency between [80,81] in conclusions for Sweden, a country in Cluster 4, is precisely due to the complexity in the epidemic situation and policy implementation in Cluster 4 compared to other clusters.

Additional clustering methods, such as the agglomerative hierarchical clustering and the density-based spatial clustering with noise (see Figs. S1 and S2 in Supporting Information), confirm these findings and underscore the robustness and reliability of our clustering approach.

### 3.2 Control measures have significant impacts on the epidemic dynamics

To effectively describe the transmission dynamics of the first wave, we introduce six dynamic features, including *Q*_100_*/N*, *R*_100_*/N*, *D*_100_*/N*, *t*_1*/*2_, *t*_*lag*_, and *k*_*app*_. Detailed in the section of Materials and Methods, their values are extracted from the WHO data (see data file 4 in Supplementary).

**Fig. 3:**
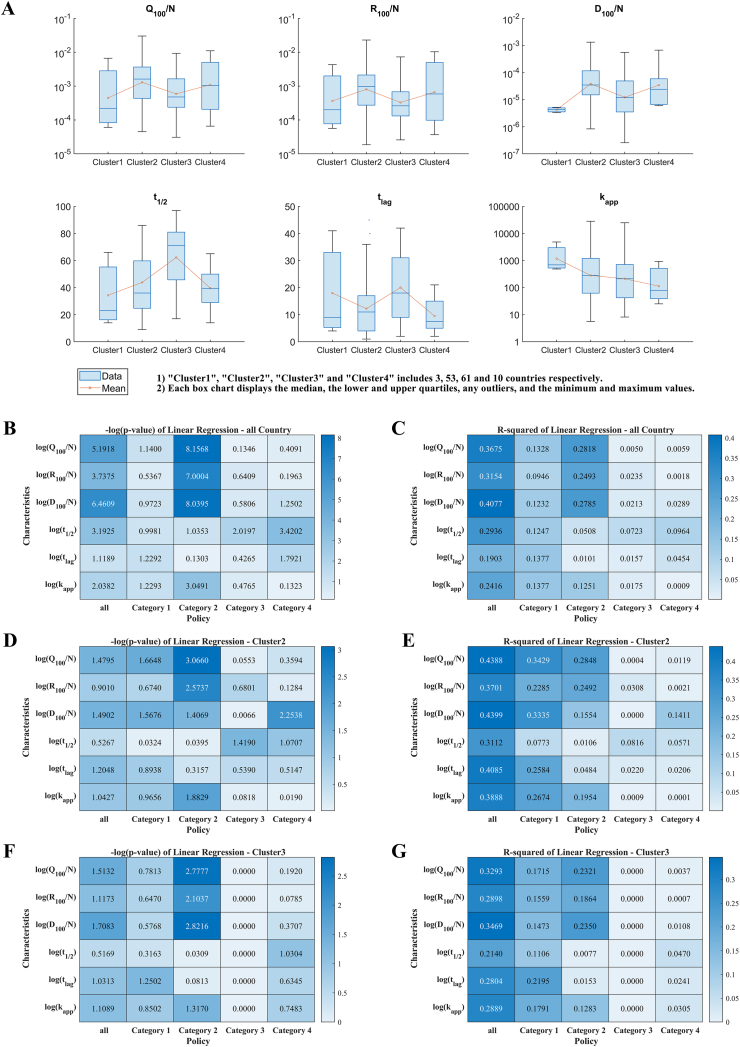
Impacts of control measures on the spreading dynamics of COVID-19 epidemics through the multiple linear regression analysis. (A) Variations of 6 epidemic dynamical features among countries belonging to different clusters are illustrated through box plots. After taking base-10 logarithmic transformation, their linear correlations with control measures in different categories for (B–C) all nations or nations in (D–E) Cluster 2 and (F–G) Cluster 3 are highlighted through (B,D,F) the p-values (the logarithm to be exact) and (C,E,G) R-square values separately. Notice the p-values and R-square values are not applicable to Category 3 for nations in Cluster 3 in (F) and (G).

As illustrated in Fig. 3A, the half time *t*_1*/*2_ and the spreading rate *k*_*app*_ depend on the control strength over countries in the first three clusters in a monotonic manner. The non-monotonic dependence for the cumulative confirmed, cured and death cases on the 100th day could be attributed to the fact the epidemics in those less developed countries/territories are still out of control on the 100th day since its outbreak. The extraordinarily long lag-time for countries in Cluster 1 shows that the spreading of COVID-19 virus has been suppressed at a very low level due to the extremely strict containing measures taken by these three countries.

A more quantitative analysis is carried out by adopting the multiple linear regression. Based on the p-values and R-square values summarized in Fig. 3B–G, several general conclusions are reached, *i.e.*

• Except for *t*_*lag*_, the linear correlations between each dynamical feature and all control measures are statistically significant when considering all nations.
• The impacts of the control measures in Category 2 on the cumulative con-firmed/cured/death cases on the 100th day *Q*_100_*/N, R*_100_*/N, D*_100_*/N* and the spreading rate *k*_*app*_ are statistically significant. These findings reveal the importance of keeping track of suspected cases, who have close contact with confirmed cases, for maintaining the epidemic under control. According to Ref. [10], the impact of contact tracing is more prominent than that of travel restriction or contact reduction.
• For all countries, the p-values for the linear correlations between the half time/lag time and measures in Category 4, the half-time and measures in Category 3 are all less than 0.05, meanwhile their corresponding R-square values are relatively small too. This fact reveals the intrinsic diversity and complexity of the spreading dynamics of COVID-19 epidemics. Solely raising up the control strength of single measure (or few measures) may not be as effective as expected.
• For countries in Cluster 2, notable correlations exist between *Q*_100_*/N* and measures in Category 1, *D*_100_*/N* and measures in Categories 1 and 4 with p-values all smaller than 0.05, while such correlations are absent in Cluster 3. This result suggests that strengthening the public control, like school and workplace closures, can be effective only when the epidemic is still under control. So is the increase of medical resources for reducing the death cases.

Our above findings based on the multiple linear regression agree with previous reports in quality. A study [82] on 11 countries in Europe by means of the semi-mechanical combined Bayesian stratification model showed that blockade control has a great impact on reducing transmission. Francisco et al. [17] summarized 34 literature and concluded that school closure is the most effective NPI, followed by workplace closure, closure of businesses and venues, and banning public events. Public awareness campaigns and the requirement to wear masks are also effective in controlling the pan-demic while being less disruptive to the population than other NPIs. By combining four methods, namely Case-control analysis, Step function approach to Lasso time-series regression, Random forests, and Transformers, on three policy datasets, Haug et al. [80] found that the most effective national measures include curfews, lockdown, and long-term closures and restrictions on gathering places with a large number of people. By analyzing 41 countries using a Bayesian hierarchical model, Brauner et al. [13] found that closing schools and universities is very effective in reducing transmission. Meanwhile, targeted closures of restaurants, bars, nightclubs and other businesses with a high infection risk that have face-to-face contact are not so useful. The effectiveness of shutting down most non-essential businesses that provide personal services is moderate. The relation between the above studies and ours is that the countries mentioned above belong to our Cluster 2, while the policies mainly belong to our Category 1.

### 3.3 The SEIR-QD model exhibits a statistically significant mediation effect

To delve deeply into the quantitative impacts of control measures, we refer to predictive mathematical models. To be specific, the SEIR-QD model (Eq. (3a)-(3g)), which acts as a representative of general compartment model and has been shown to be a suitable model for studying COVID-19 epidemics [68,69], is considered here.

**Fig. 4:**
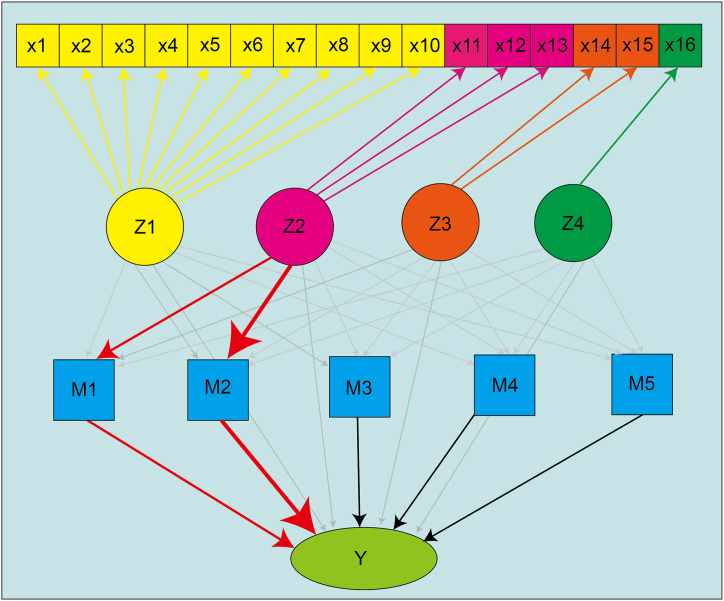
Illustration on the mediation effect of the SEIR-QD model. x1−x16 denote 16 control measures summarized in Table 1, which have been clustered into four categories Z1 −Z4. M1 −M5 represent the logarithm of five coefficients in the SEIR-QD model, and the scalar Y takes each of the six dependent variables, {log(Q100/N), log(R100/N), log(D100/N), log(t1/2), log(*t*_*lag*_), log(*k*_*app*_)}, in sequence. The pathways exhibiting statistically significant mediation effects are highlighted in red. Note it exactly reflects the structure equation we adopted in performing the mediation analysis. (For interpretation of the references to color in this figure legend, the reader is referred to the Web version of this article.)

The mediation analysis using the structural equation modeling (see Sect. 2.2) con-firms the pivotal mediating role of the SEIR-QD model between control measures and epidemic dynamics (see Fig. 4). Our main findings are summarized as follows.

• The total mediation effect of the SEIR-QD model is statistically significant for four epidemic dynamical features, i.e. the normalized cumulative numbers of con-firmed/cured/death cases on the 100th day *Q*_100_*/N, R*_100_*/N, D*_100_*/N* and the spreading rate *k*_*app*_*.*
• The infection rate *β* emerges as a critical mediator, particularly for Category 2 measures influencing the four dynamic features, as illustrated through red arrows in Fig. 4. This fact can be clearly understood, since more frequent nucleic acid tests and continued tracking of suspected cases allow for a shorter time to identify these infected cases and isolate them from normal people, and thus effectively reduce the infection rate *β.*
• The mediation effect from control measures in Category 2 to *Q*_100_*/N* through the protection rate *α* is also statistically significant, validating the necessity of including the protection rate into the SEIR model to accurately represent the impacts of control measures.

These findings are consistent with the report of Zhou et al. [30], which emphasized the importance of contact tracing and testing during the outbreaks of Delta variant in Xi'an, Yangzhou and Guangzhou in China. Pei et al. [83] also found that improving the testing and contact tracing capabilities is critical before the COVID-19 rebound begins.

### 3.4 The SEIR-QD model allows a robust quantitative evaluation on the impacts of control measures

The SEIR-QD model provides a quantitative way to estimate the impacts of various control measures and their implementation intensities on the transmission dynamics of COVID-19 epidemics. Here we establish a close relation between the control measures and parameters in the SEIR-QD model.

• Control measures in Category 1 predominantly affect the protection rate *α* and the infection rate *β.* Enhancing control measures belonging to this category typically lead to an increased *α* and a reduced *β.*
• Control measures in Category 2 impact not only the infection rate *β* but also the quarantine rate *δ*, since intensifying nucleic acid testing and contact tracking will shorten the time to identify infected cases, making their isolation more quickly. This is supported by the linear correlation analysis and the mediation analysis too.

• Statistically significant linear correlations exist between control measures in Categories 3 & 4 and the mortality rate *κ.* Enhancing medical resources generally leads to a reduced mortality rate.

**Fig. 5:**
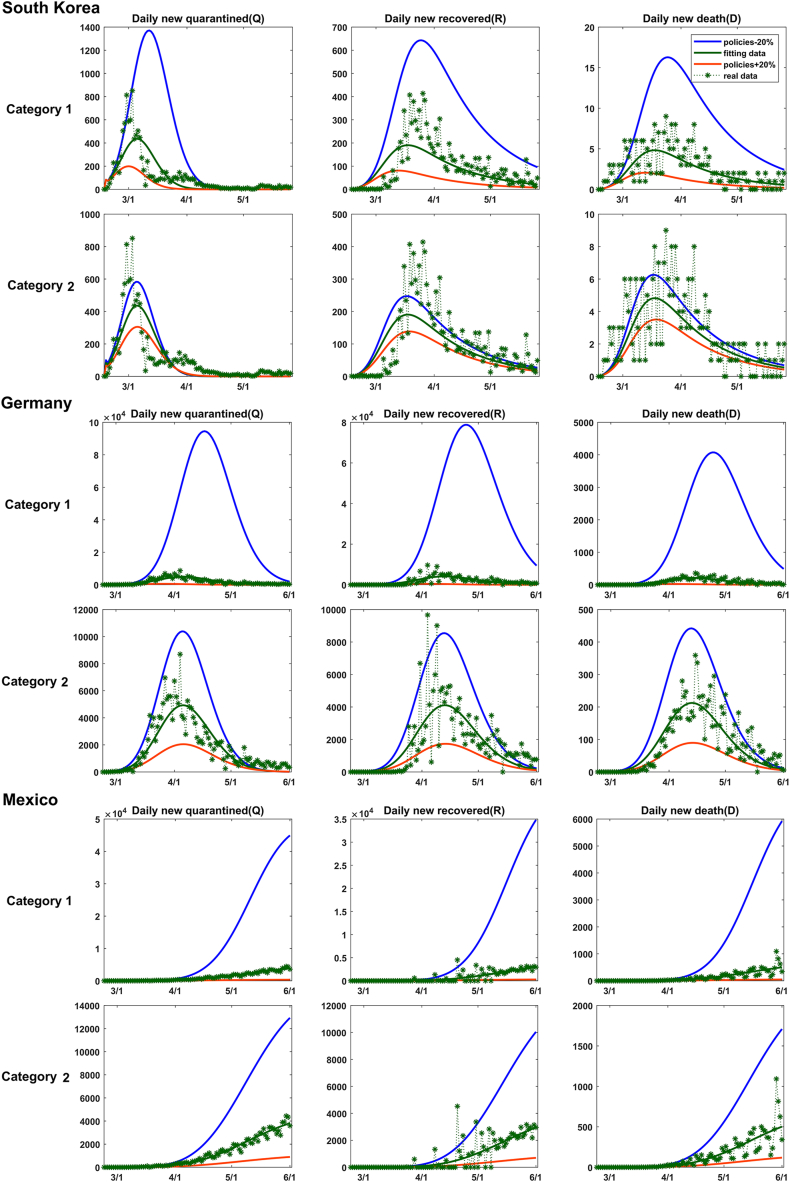
Influence of control measures in Category 1 & 2 evaluated through predictions of the SEIR- QD model. Here, South Korea in Cluster 1, Germany in Cluster 2 and Mexico in Cluster 3 are taken as the representative in each corresponding cluster. In comparison with base lines (real data: green stars with dashed lines, model fitting: green solid lines), parameters α and β are changed by +20 % (blue lines) and −20 % (red lines) to mimic the influence of control measures in Category 1. So are the parameters β and δ for measures in Category 2. Meanwhile, the rest parameters are kept at their default values. From these plots, we can reach a consistent conclusion among a majority of countries under study that the impacts of policies in Category 2 are relatively weaker than those in Category 1 on the epidemic dynamics. (For interpretation of the references to color in this figure legend, the reader is referred to the Web version of this article.)

Adjusting parameters corresponding to control measures in each category by ±20 % with respect to their default values (while keeping the rest parameters unchanged) reflects the diverse impacts of policies. As depicted in Fig. 5, taking South Korea (belonging to Cluster 1), Germany (Cluster 2), and Mexico (Cluster 3) as representative examples in each cluster, their epidemic dynamics exhibit varied dependencies on the control measures. For instance, a 20 % relaxation in control measures results in minor changes in South Korea but significant impacts in Mexico, and Germany in-between. Additionally, the influence of control measures in Category 2 is weaker than that of measures in Category 1. These observations accurately reflect the varied situations in these countries. For further insights, please refer to Supporting Information for data on other representative countries.

**Fig. 6:**
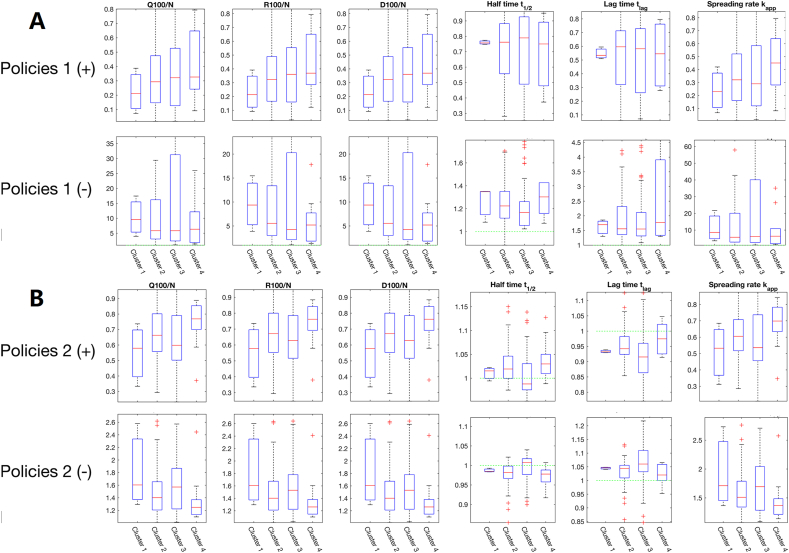
Impacts of control measures evaluated through the sensitivity analysis on the SEIR-QD model. The box plots show the fold changes in each of the six dynamical features for all countries belonging to Cluster 1 to 4 respectively. (A) Parameters α and β are changed by ±20 % to mimic the effects of measures in Category 1. Meanwhile, all other parameters are kept at their default values. For comparison, countries in four clusters are illustrated separately. Their corresponding fold changes are indicated through the vertical axes. So are the parameters β and δ for Category 2 in (B).

In addition, the global sensitivity analysis is performed (see Fig. 6). As reflected by the normalized cumulative numbers of confirmed/cured/death cases on the 100th day, *Q*_100_*/N, R*_100_*/N, D*_100_*/N*, tightening measures in Category 1 seems to be more effective than those in Category 2 albeit at a higher cost. Notably, the impact of measures in Category 1 on *Q*_100_*/N* demonstrates a monotonic trend across clusters, with the strongest effect in Cluster 1 and the weakest in Clusters 3 and 4. Contrarily, non-monotonic behaviors are observed for impacts of control measures in Category 2 among four country clusters. The average influence of nucleic acid testing and keeping tracing of suspected cases for countries in Cluster 3 is more apparent than expected. Interestingly, control measures in Category 1 and 2 have opposing effects on the half time, suggesting that testing and contact tracing primarily prolongs the peak time of the epidemic. In summary, the policy impacts on the transmission dynamics of COVID-19 are significantly different among four clusters.

Besides the compartment model, we also conduct the multiple linear regression and a nonlinear regression by using the multilayer perceptron (MLP). The MLP model demonstrates a high degree of fit with the real epidemic data, outperforming the multiple linear regression model that shows only a moderate efficacy. However, both linear and nonlinear regression models fail to yield consistent results during the sensitivity analysis (see Figs. S4, S10, S11 in SI). Consequently, these models appear unsuitable for assessing the impacts of policy measures on the dynamics of COVID-19 epidemics.

## 4 Conclusion and discussion

In the field of epidemiology, a crucial issue is to what extent the implemented control measures and their respective strength can influence the epidemic trend. It has sparked significant controversy, particularly during each epidemic caused by infectious diseases such as influenza, SARS, Ebola, and many others. In this paper, by performing comprehensive analyses based on the officially reported epidemic data of 127 countries/territories and 16 concrete control measures taken by each country during the first wave of COVID-19 epidemic, the remarkable impacts of control measures and their effectiveness are elucidated quantitatively. In particular, the mediation effect of compartment models, represented by the SEIR-QD model in the current study, is analyzed with respect to real epidemic data and is proved to play a statistically significant role. This, to the best of our knowledge, is the first of its kind in the field of epidemiology. And our findings confirm the necessity and significance of compartment models during the study of epidemics from a new perspective.

To help policy makers and researchers implement more effective prevention strategies tailored to their respective national contexts, we summarize the key findings of the study below.

• During the first wave of COVID-19 pandemic, there is a dramatic distinction in the control measures and their implementation strengths among major countries/territories in the world, which largely affects the spreading speed and infected population size in each country.
• Several key dynamical features, like the normalized cumulative numbers of con-firmed/cured/death cases on the 100th day, the half time and the apparent spreading rate, show statistically significant linear correlations with the overall control measures.
• The SEIR-QD model, especially the infection rate *β* and protection rate *α*, exhibits a statistically significant mediation effect between the control measures and dynamical features for epidemics. In particular, the mediation effect along the pathway from control measures in Category 2 to four dynamical features – *Q*_100_*, R*_100_*, D*_100_ and *k*_*app*_ through the infection rate *β* is the most prominent. This fact highlights the importance of nucleic acid testing and keeping tracing suspected cases to contain the epidemics under control.
• The compartment models, in particular the SEIR-QD model in the current study, allow a robust quantitative evaluation on the policy impacts. In contrast, no consistent result could be reached by either the multiple linear regression or neural-network-based nonlinear regression.

Apparently, our current study is far from complete. Firstly, the COVID-19 epi-demic is an exceedingly complex process, which means numerous other factors may come into play alongside control policies. For example, the movement of people across borders, the presence of asymptomatic cases, the emergence of new mutant types of coronavirus and so on, make it almost impossible to keep a country free from infection, no matter what kinds of control measures are implemented. Furthermore, even with a strong willing, many countries/territories are facing with significant challenges in implementing high-level controls on the public gathering, workplace closure, etc., due to both economic and social issues. These facts are totally ignored in the current study to a great pity. We also constrain our analyses on the first-wave data of COVID-19 epidemic, which means the more fruitful phenomena appeared in the second and following waves of COVID-19, the temporal changes in the control measures of each country in response to the progressing of epidemics have not been taken into consideration yet.

It is also a critical issue whether the results we obtained in the current article are robust and reliable. To avoid over-interpretation, the data are aggregated, the policies are grouped and the countries under study are clustered. Almost all statements are reached in the statistical sense over plenty of countries with similar situations. Meanwhile, uncertainty quantification on the accuracy and robustness of model parameters, as well as their consequence on the predicted epidemic dynamics have been carried out too (see Figs. S6, S7, S8 in SI). Our preliminary results show that there is no apparent contradiction to the general conclusions in this work. In the future, we hope to carry out more comprehensive analyses by utilizing alternative advanced statistical approaches and machine learning based methods [80], to provide a better understanding on the issues discussed in the current paper.

## Funding statement

This work was supported by the 10.13039/501100012166National Key R&D Program of China (Grant No. 2023YFC2308702), the 10.13039/501100001809National Natural Science Foundation of China (11801020, 12205135, 12301617), 10.13039/501100021171Guangdong Basic and Applied Basic Research Foundation (2023A1515010157), the 10.13039/501100003392Natural Science Foundation of Fujian Province of China (2020J05172), Startup Research Funding of 10.13039/501100009696Minjiang University (mjy19033), Special Pre-research Project of 10.13039/501100003444Beijing University of Technology for Fighting the Outbreak of Epidemics.

## Data availability statement

The code is available at https://github.com/DLforS/ControlMeasuresAndCOVID-19Epidemics.

## CRediT authorship contribution statement

**Dongyan Zhang:** Writing – review & editing, Writing – original draft, Data curation. **Wuyue Yang:** Writing – review & editing, Writing – original draft, Data curation. **Wanqi Wen:** Writing – review & editing, Writing – original draft, Data curation. **Liangrong Peng:** Writing – review & editing, Writing – original draft. **Changjing Zhuge:** Writing – review & editing, Writing – original draft, Project administration, Investigation, Data curation, Conceptualization. **Liu Hong:** Writing – review & editing, Writing – original draft, Project administration, Investigation, Data curation, Conceptualization.

## Declaration of competing interest

The authors declare the following financial interests/personal relationships which may be considered as potential competing interests:

Liu HONG reports financial support was provided by National Key R&D Program of China. Changjing Zhuge reports financial support was provided by 10.13039/501100001809National Natural Science Foundation of China. Liangrong Peng reports financial support was provided by 10.13039/501100001809National Natural Science Foundation of China. Liu HONG reports financial support was provided by 10.13039/501100021171Guangdong Basic and Applied Basic Research Foundation. Liangrong Peng reports financial support was provided by 10.13039/501100003392Natural Science Foundation of Fujian Province of China. If there are other authors, they declare that they have no known competing financial interests or personal relationships that could have appeared to influence the work reported in this paper.

## Abbreviations

COVID-19: Corona Virus Disease 2019
MLP: MultiLayer Perceptron
NPI: Non-pharmaceutical intervention
OxCGRT: Oxford COVID-19 Government Response Tracker
SEM: Structural Equation Modeling
WHO: World Health Organization
