# Supplementary material for: A data-driven analysis on the mediation effect of compartment models between control measures and COVID-19 epidemics
Source: Heliyon. 2024 Jun 29;10(13):e33850. doi: 10.1016/j.heliyon.2024.e33850 (PMC11283110; doi:10.1016/j.heliyon.2024.e33850)
Supplement: Multimedia component 5 [file mmc5.pdf]

Supporting Information

Dongyan Zhang[1,2*],Wuyue Yang[3*] , Wanqi Wen[1], Liangrong Peng[4] , Changjing Zhuge[2†] , and Liu Hong[1†]

[1]School of Mathematics, Sun Yat-Sen University, Guangzhou, 510275, PR China.
[2]Department of Mathematics, School of Mathematics, Statistics and Mechanics, Beijing University of Technology, Beijing, 100124, PR China.
[3]Beijing Institute of Mathematical Sciences and Applications, Beijing, 101408, PR China.
[4]College of Mathematics and Data Science, Minjiang University, Fuzhou, 350108, PR China.

List of Figures:

1. Hierarchical tree for country clustering.
2. Country clustering by DSCAN method.
3. Linear correlations among six dynamical features, five coefficients of SEIR-QD model and sixteen control measures for all 127 countries.
4. Linear correlations between true values of six dynamical features and predictions by multiple linear regression model, MLP and SEIR-QD model.
5. Performance of SEIR-QD model for eight representative countries.
6. Uncertainty quantification on SEIR-QD model.
7. Sensitivity analysis by SEIR-QD model for eight representative countries.
8. Sensitivity analysis by SEIR-QD model for all 127 countries.
9. Impacts of measures in Category 1 & 2 on COVID-19 epidemics in China, Canada and Colombia evaluated by SEIR-QD model.
10. Sensitivity analysis by MLP.
11. Sensitivity analysis by multiple linear regression model.

[*]These authors have contributed equally to this work.
[†]Correspondence authors: CZ: zhuge@bjut.edu.cn, LH: hongliu@sysu.edu.cn

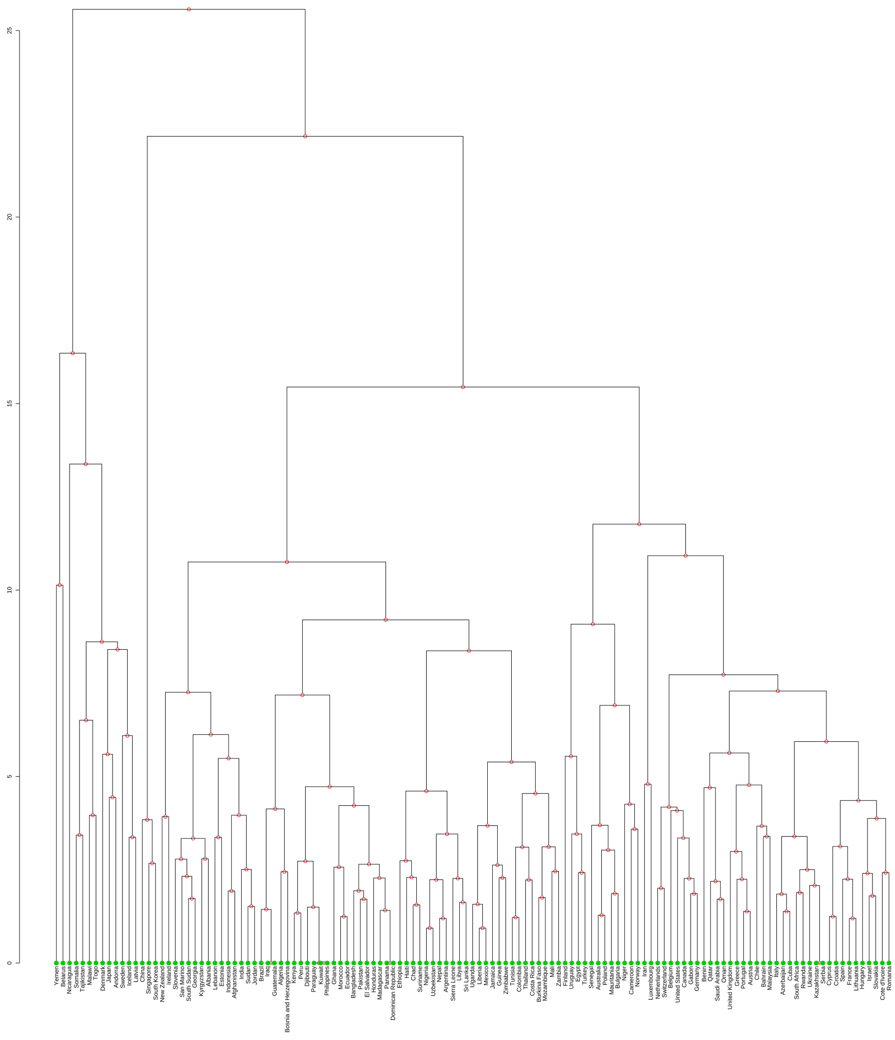

Figure S1: Hierarchical tree by agglomerative hierarchical clustering methods based on policy grades of a country.

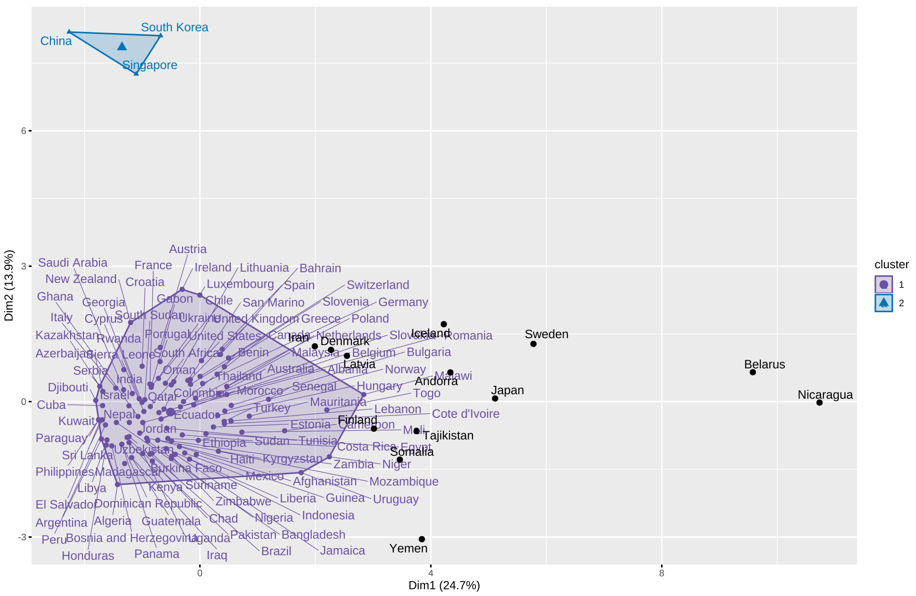

Figure S2: Country clustering by density-based spatial clustering and application with noise method based on policy grades of a country.

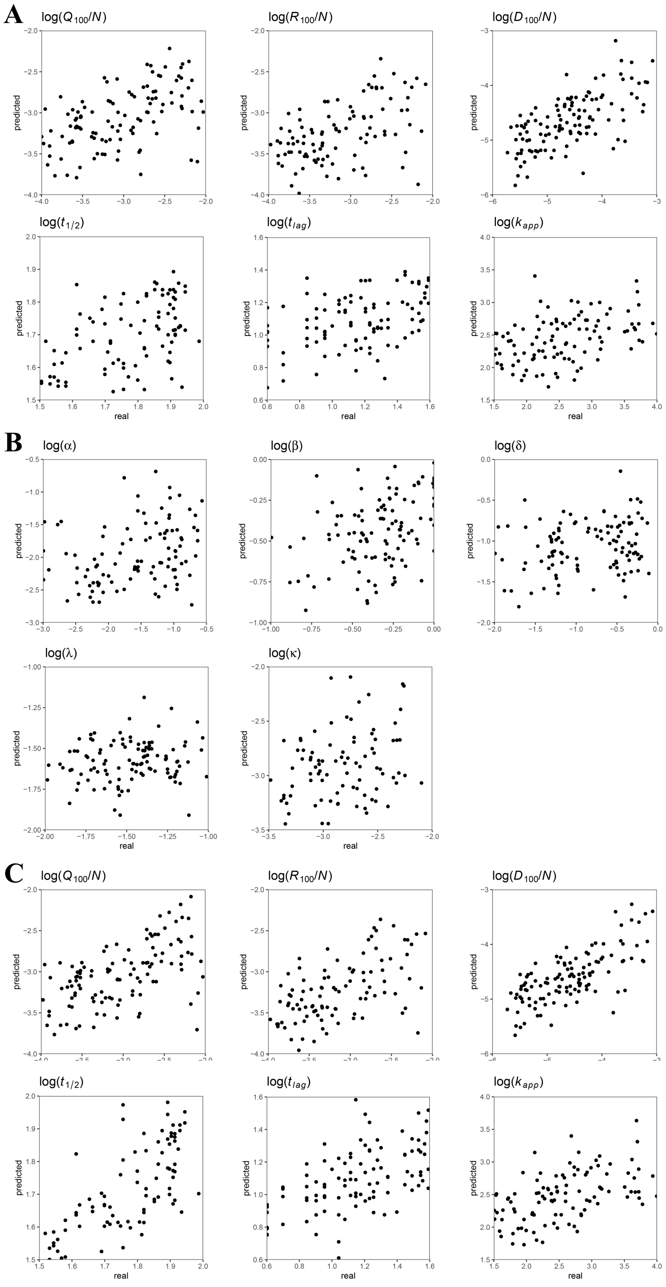

Figure S3: Illustration on the linear correlations between predicted values and their true values. The logarithms of (A) six dynamical features are predicted based on sixteen control measures by using the multiple linear regression method. So are (B) the logarithms of five model coefficients based on sixteen control measured and (C) the logarithms of six dynamical features based on five model coefficients.

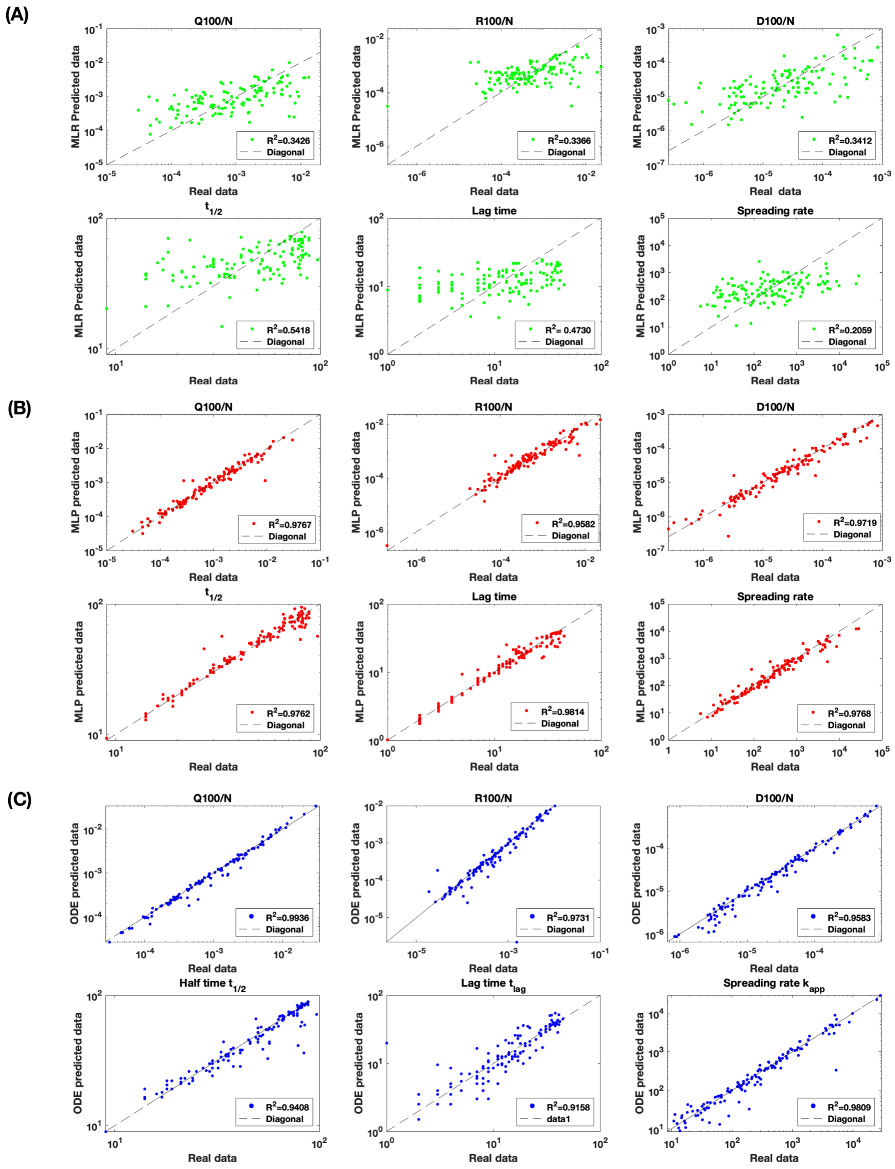

Figure S4: Linear correlations between true values of six dynamical features derived from the WHO epidemic data and best predictions by the multiple linear regression (MLR) model (green dots), MLP (red dots) and SEIR-QD model (blue dots).

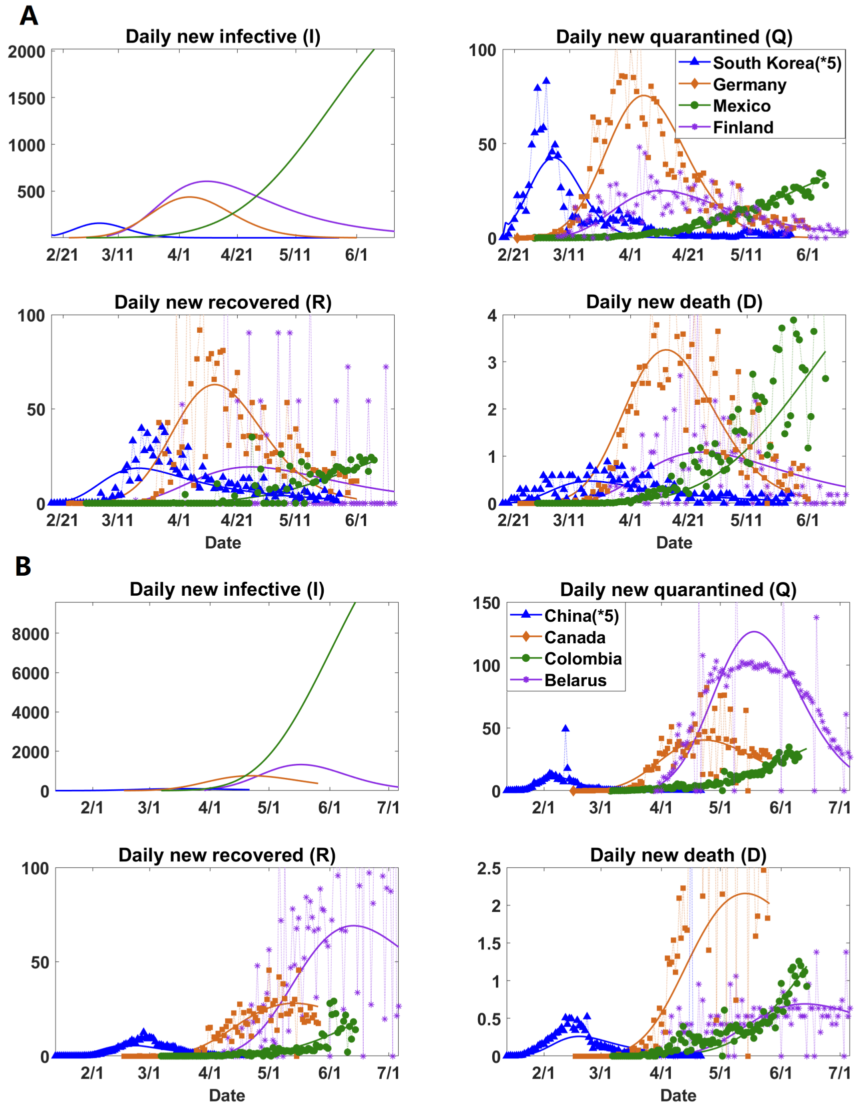

Figure S5: Comparions on the predictions of SEIR-QD model with officially reported epidemic data by WHO. For each cluster, two representive countries are selected. Countries are distinguished by their colors. We have purple for Finland, blue for South Korea, brown for Germany and green for Mexico in (A), and purple for Belarus, blue for China, brown for Canada and green for Colombia in (B). Solid lines represent the predicted dynamics by the SEIR-QD model, while the dotted dashed lines represent the real data. The vertical axis shows the number of cases per a million of population.

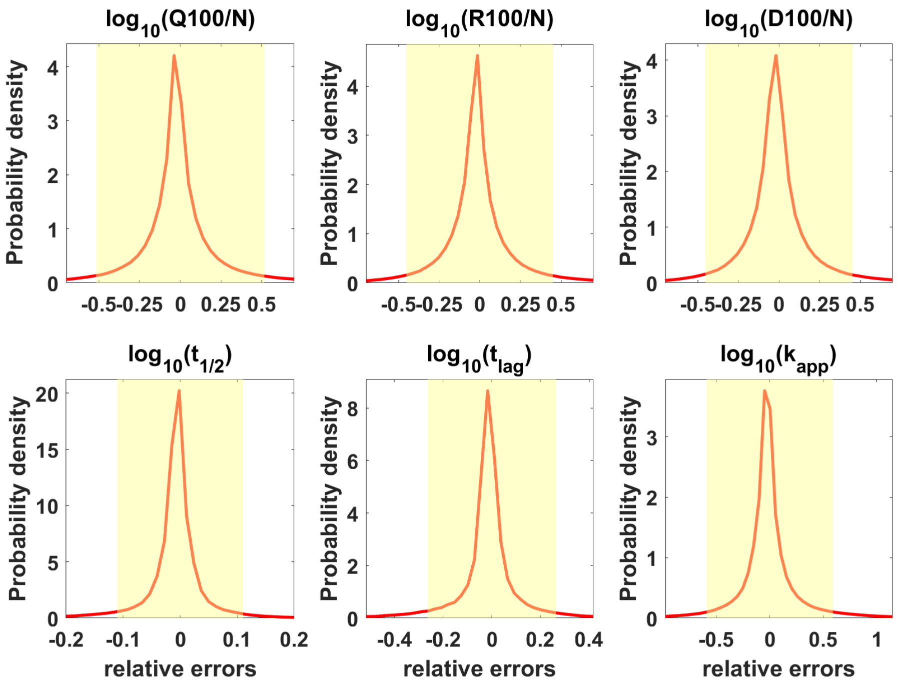

Figure S6: Distributions of the relative errors on predicitons of the SEIR-QD model. The five model coefficients are perturbed randomly and independently by up to 5% with respect to their default values. The corresponding changes in model predictions are measured through relative errors of the logarithm of six dynamical features. 10000 times caluclations are performed independently, which give the distributions in each subplot. The 95%a confidence intervals are marked in yellow.

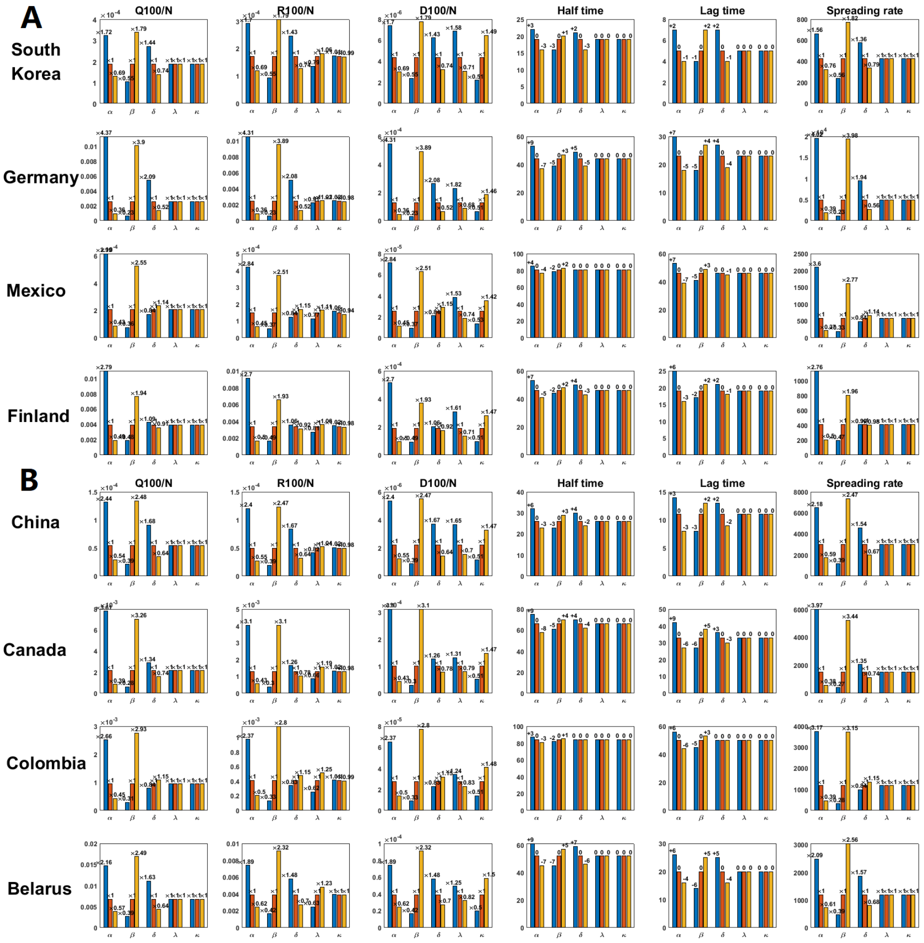

Figure S7: Sensitivity analysis on six epidemic dynamical features affected by the coefficients of SEIR-QD model. For each cluster, two representive countries are selected. Blue and yellow bars represent perturbations of the marked coefficient by $\mp 20\%$ for α, β, δ and $\mp 50\%$ for λ, κ with respect to the base values (red bars).

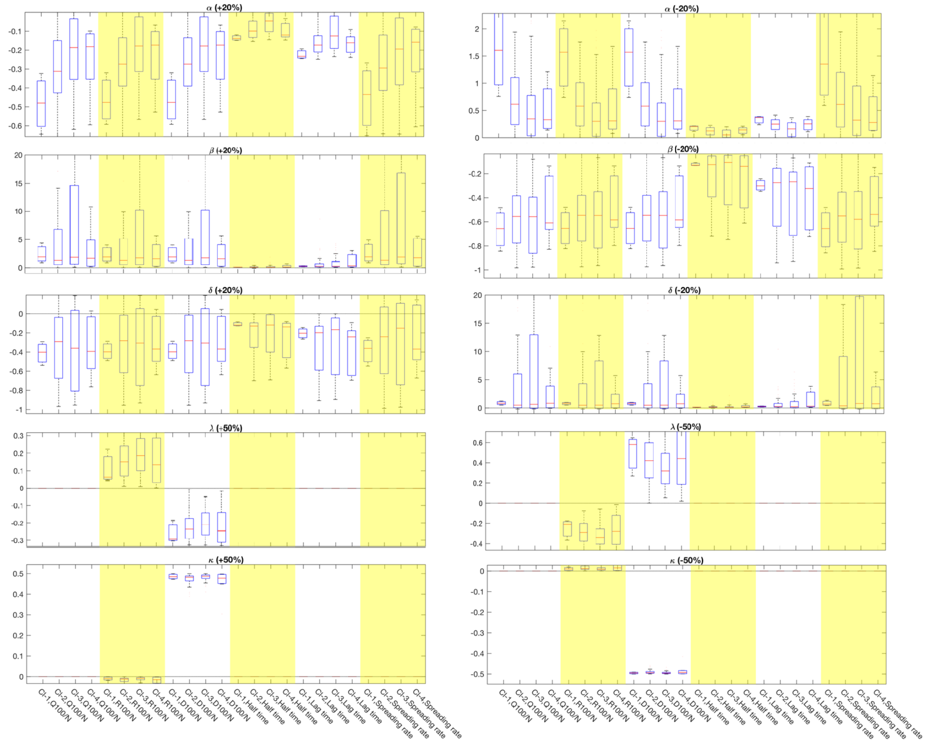

Figure S8: Sensitivity analysis on six epidemic dynamical features affected by five coefficients of SEIR-QD model for all 127 countries. Their relative changes are marked for countries in Cluster-1 to Cluster-4 separately.

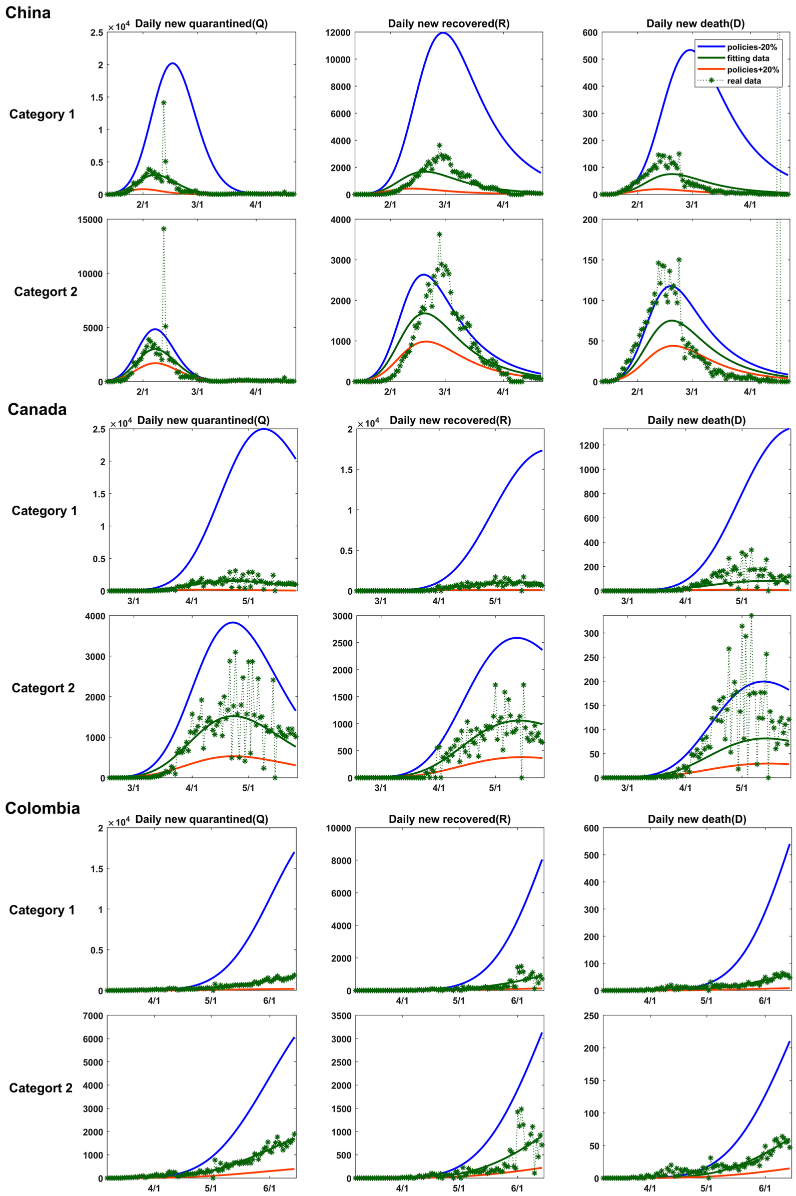

Figure S9: Impacts of control measures in Category 1 & 2 on the COVID-19 epidemics evaluated through the SEIR-QD model. China, Canada and Colombia are chosen as representatives for countries in Clusters 1 to 3. In comparison with base lines (green stars with dashed lines for real data and green solid lines for model fittings), parameters α and β are changed by $+20\%$ (blue lines) and -20% (red lines) to mimic the influence of control measures in Category 1. So are parameters β and δ for measures in Categories 2.

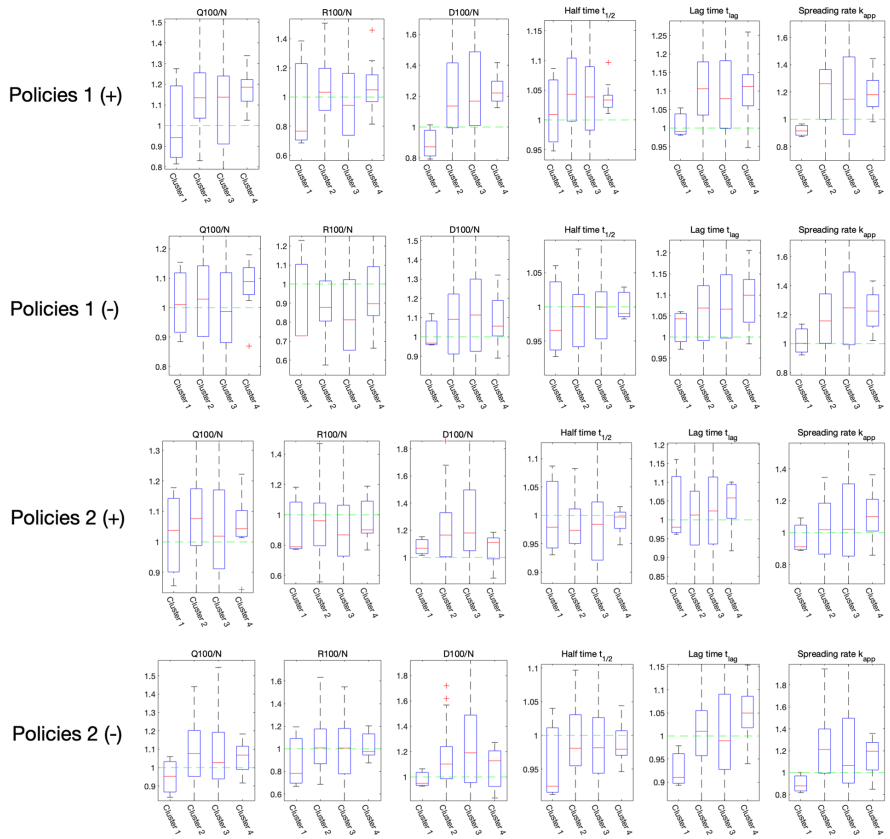

Figure S10: Predictions of MLP on the influence of control measures in Categories 1 & 2 on six epidemic dynamical features for all 127 countries. Their relative fold changes are marked for countries in Cluster-1 to Cluster-4 separately.

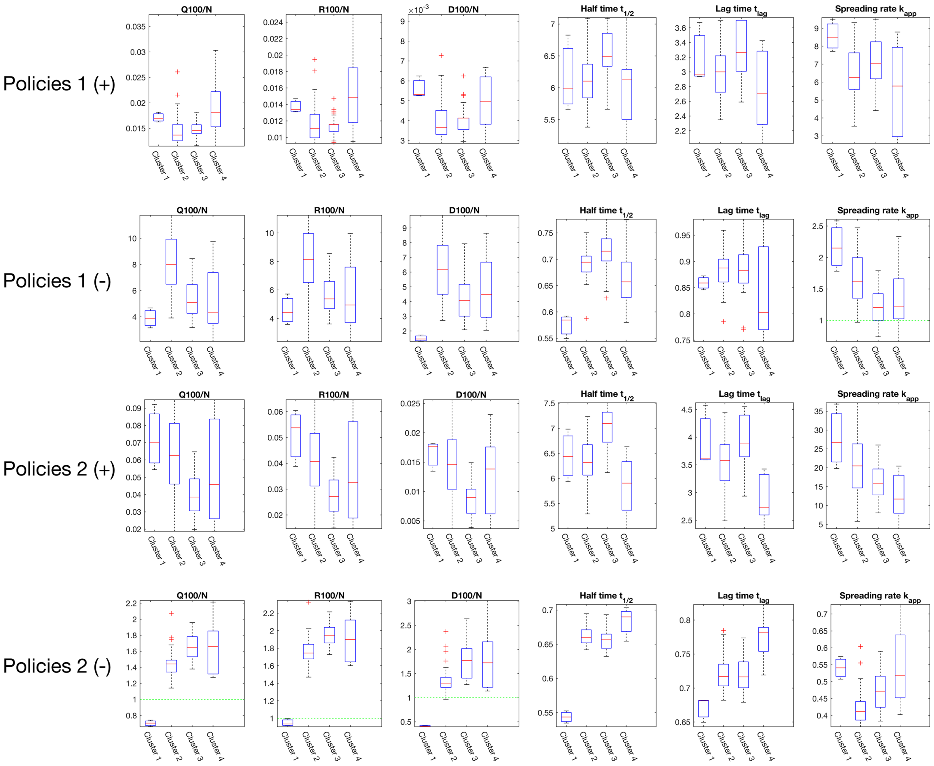

Figure S11: Predictions of the multiple linear regression model on the influence of control measures in Categories 1 & 2 on six epidemic dynamical features for all 127 countries. Their relative fold changes are marked for countries in Cluster-1 to Cluster-4 separately.
